# Evidence against efficient spontaneous disassembly of prions into small oligomers

**DOI:** 10.1016/j.jbc.2025.110411

**Published:** 2025-06-21

**Authors:** Daniel Shoup, Andrew G. Hughson, Brent Race, Parvez Alam, Daniel Dulebohn, Suzette A. Priola, Byron Caughey

**Affiliations:** 1 Laboratory of Neurological Infections and Immunity, Rocky Mountain Laboratories, Division of Intramural Research, National Institute of Allergy and Infectious Diseases, National Institutes of Health, Hamilton, Montana, USA; 2 Research Technologies Branch, Rocky Mountain Laboratories, Division of Intramural Research, National Institute of Allergy and Infectious Diseases, National Institutes of Health, Hamilton, Montana, USA

**Keywords:** amyloid, prion, prion disease, oligomer, fibril

## Abstract

Infectious prion assemblies must fragment to replicate, spread, and trigger disease. However, the extent to which various types of amyloid fibrils fragment on their own *versus* being driven by other cellular processes is unclear. In the case of highly infectious, tissue-derived prion (PrP^Sc^) preparations, over 40 years of previous studies have yielded starkly contradictory indications on this question. Many have reported high stability of PrP^Sc^ multimers in even strong detergents. However, others using nondisinfecting detergents and size-exclusion chromatography combined with light scattering measurements have described complete spontaneous disassembly into dimeric-tetrameric units. In attempting to replicate the latter experiments, we determined that PrP^Sc^ size-exclusion elution behavior was dominated by binding to the column matrix, not particle size. The light scattering behavior of fractions containing PrP^Sc^ was dominated by the coelution of detergent micelles similar in size to hypothetical PrP^Sc^ dimers-trimers. Furthermore, sedimentation velocity centrifugation and electron microscopy indicated that most detergent-treated PrP^Sc^ particles remained larger than 70-mers. When added to live cells that lacked prion protein and were therefore incapable of new PrP^Sc^ assembly, most PrP^Sc^ remained in the form of large multimers for ≥24 h, confirming substantial stability in a cellular model. Thus, we found no evidence that the much larger assemblies that predominate in brain homogenates or purified PrP^Sc^ preparations fragment spontaneously into small oligomers. Moreover, our identification of prion-associated size-exclusion chromatography artifacts reconciles previously disparate reports about prion disassembly.

In the many diseases that feature prion-like propagation of various types of amyloid fibrils, it is important to understand not only how the fibrils grow, but also how they can be fragmented to account for both exponential increases in fibril number and spreading within and between cells. A closely related issue is the relative stabilities of pathological parallel in-register intermolecular β-sheet (PIRIBS) assemblies across a wide range of sizes from small oligomers to fibrils and plaques.

Using rodent-adapted prion strains as highly infectious examples of pathological amyloids, we sought to gain insight into these issues. Prion protein (PrP) based prion diseases are untreatable, lethal, and transmissible neurodegenerative disorders. The transmissible agents (prions or PrP^Sc^) of diseases such as scrapie in sheep or Creutzfeldt-Jakob disease in humans are composed primarily of misfolded assemblies of the host’s PrP molecules (1, 2, 3). Whereas the normal properly folded form of PrP (PrP^C^) has largely disordered and α-helical N- and C-terminal domains, respectively (4), the infectious (prion) forms have high β-sheet content and lack α-helical secondary structure (1, 5, 6, 7, 8, 9, 10, 11, 12). The structures of several highly infectious natural (12) and experimental rodent-adapted (1, 8, 9, 10, 11) prion strains have been solved at near atomic resolution by cryo-EM, revealing amyloid fibrils with ordered cores comprised of PrP monomers (*i.e.*, polypeptide chains) aligned along the fibril axis *via* a serpentine series of loops and parallel, in-register intermolecular β-sheets. Many pathological amyloids have PIRIBS architectures, which can mechanistically rationalize the faithful propagation of specific disease-associated conformers and prion strains (13, 14, 15, 16, 17, 18).

Although PrP^Sc^ fragmentation is needed to account for both exponential increases in prion titer (*e.g.* (19)) and the neuroanatomical spreading of prions to the point where they cause disease, the fragmentation mechanisms and the size range of the resulting fragments are unclear. Prion fragmentation might either be spontaneous, *i.e.*, due to inherent instabilities in their structures, or the result of other physiological activities such as disaggregases, membrane dynamics, or other cellular processes (20).

Many previous studies have attempted to assess prion disassembly and characterize the minimal infectious units, with starkly conflicting interpretations. A few studies, beginning with Stanley Prusiner’s 1982 paper (21), have concluded that PrP^Sc^ can almost completely disassemble into dimers-to-tetramers in detergents. Similarly, a recent study describes nearly complete disassembly within 4 h in brain homogenates (BH) treated with dodecylmaltoside (DDM) and sarkosyl without diminishing prion infectivity (22). In contrast, many others (*e.g.* (23, 24, 25, 26, 27, 28)), including a different Prusiner study (29), have reported evidence that the vast majority of detergent treated PrP^Sc^ particles remain much larger.

In hopes of evaluating the size and structure of the minimum infectious PrP^Sc^ unit, we have attempted to isolate small oligomers as recently described (22). However, we found strong evidence that studies describing the efficient detergent-induced disassembly of PrP^Sc^ into small oligomers were likely misled by size-exclusion chromatography (SEC) artifacts. We also failed to find evidence of extensive prion disassembly in live cells. With these observations, we offer an explanation for the long-standing discrepancies in the literature about the extent to which prions fragment spontaneously into small oligomers that can account for prion propagation in the infected host. In a companion article (30) we also provide molecular dynamics analyses of oligomeric units of cryo-EM-derived infectious prion fibril cores *in silico* which provide evidence that, although oligomers can be relatively stable when as small as tetramers, larger oligomers up to 25-mers, at least, show no signs of spontaneous fragmentation even at elevated temperatures.

## Results

### Effects of DDM and sarkosyl treatments of 263K prions in brain homogenate

**Figure 1:**
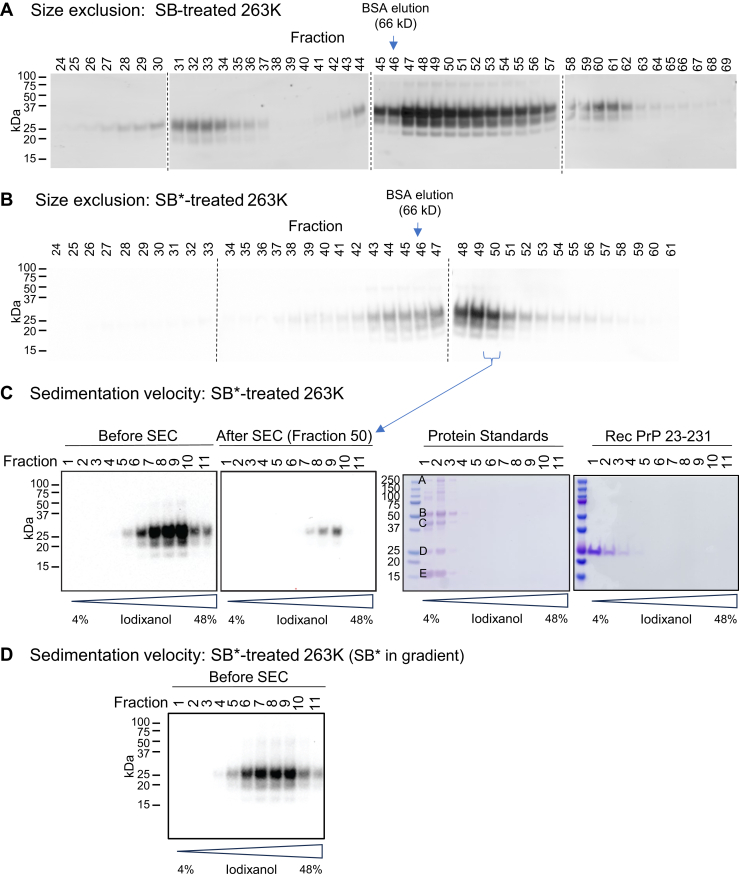
**Size-exclusion chromatography (SEC) and sedimentation velocity (SV) centrifugation of SB- and SB∗-treated 263KprionBH (pretreated with PK).***A*, anti-PrP western blots of SEC fractionation of SB-treated hamster 263K BH. *Arrow*: the fraction at which 66 kDa BSA eluted on this column. Positions of SDS-PAGE MW markers are shown on the *left*. *Dashed lines* indicate borders between individual blots. Similar elution profiles were obtained from six additional independent 236K BH treatments and SEC separations. *B*, anti-PrP western blots of SEC fractionation of SB∗-treated 263K BH. A similar elution profile was obtained in a second independent experiment. *C*, anti-PrP western blots of SV gradient fractions (*top* to *bottom*) of the SB∗-treated 263K BH before SEC (*left*) and SEC fraction #50 from panel (*B*) (*secondpanel*). Coomassie blue-stained gels of SV fractions of gradients loaded with protein MW standards (*thirdpanel*) or recombinant PrP^C^ monomer (*right*-*most panel*). The sizes of gel MW standards are shown on the *left* in *thirdpanel*. The SV protein MW standards are as follows: A. thyroglobulin (665 kDa); B. γ-globulin large subunit (55 kDa); C. ovalbumin (44 kDa); D. γ-globulin small subunit (24 kDa); E. ribonuclease (15 kDa). Recombinant PrP^C^ is 25 kDa (*right most panel*). *D*, anti-PrP western blot of SV fractions from gradient loaded with SB∗-treated 263K BH with SB∗ in gradient medium. BSA, bovine serum albumin; SB∗, corrected SB; BH, brain homogenate; PK, proteinase K; rec, recombinant.

Our initial goal was to confirm reports of efficient prion disassembly into dimeric-tetrameric units in nondenaturing detergents. We followed a recently described protocol for solubilizing prions in proteinase K (PK)-treated BH using a solubilization buffer (SB) containing 5 mM DDM and 50 mM sarkosyl for 4 h, followed by SEC analysis on a Superdex 200 GL 10/300 column (∼13–669 kDa particle separation range) without detergent in the mobile phase (22). In the previous report, PrP eluted in two predominant peaks (called P1 and P2) that, with reference to the elution of molecular weight standards, appeared to be consistent with the predominance of PrP tetramers and dimers, respectively. In contrast, in our several attempts (*e.g.*, Fig. 1*A*), PrP elution profiles suggested the additional presence of much larger PrP multimers that eluted earlier. Similar elution profiles were obtained from six additional independent 236K BH treatments and SEC separations. For two of these SEC fractionations, we tested early, middle, and late fractions using the prion real-time quaking-induced conversion (RT-QuIC) seed amplification assay, with the late fractions eluting after 66 kDa bovine serum albumin (BSA) on the same column. All of the fractions tested contained 4 to 6 log_10_/μl of prion seeding activity (Table 1). Importantly, the seeding activity in each fraction was reduced by at least 3000-fold by filtration through 300 kDa high molecular weight cutoff ultrafilters (Table 1). These results were consistent with the predominance of PrP seeds that were >∼10-mers, including in fractions 46 to 52 that would overlap with the previously described P1 or P2 peaks ascribed to tetramers and dimers (22).

**Table 1:** Ultrafiltration and RT-QuIC of fractions from SEC of SB-treated 263K BH

	Fraction	300 kDa filtration	Log_10_ SD50/2 μl	Log drop with filtration
SEC fractions after SB treatment (Run 1)	F26	-	4.25	
		+	<0.50	≥3.75
	F46	-	6.00	
		+	<0.50	≥5.50
	F52	-	6.50	
		+	3.00	3.50
SEC fractions after SB treatment (Run 2)	F26	-	5.00	
		+	0.50	≥4.50
	F33	-	5.00	
		+	0.50	≥4.50
	F49	-	6.00	
		+	1.25	4.75
SEC fractions after SB∗ treatment	F32	-	4.25	
		+	<0.50	≥3.75
	F41	-	6.00	
		+	<0.50	≥5.50
	F48	-	6.50	
		+	3.00	3.50

Moreover, sucrose gradient- and iodixanol gradient-based sedimentation velocity (SV) centrifugations of both SB-treated BHs and a fraction corresponding to the reported P1/P2 peak (#50) showed a predominance of PrP^Sc^ particles sedimenting much faster than 15 to 665 kDa markers (the largest being the mass equivalent of a PrP^Sc^ ∼22-mer) run on contemporaneous gradients, rather than the expected PrP dimers and tetramers (Supplementary Fig. 1). We concluded overall that our solubilization treatment using SB had been much less effective than expected based on the prior report.

We consulted with authors of the previous study we were following (22), who realized that their most recently reported SB formula was incorrect; specifically, the DDM and sarkosyl concentrations should be 45 mM instead of 5 mM and 80 mM instead of 50 mM, respectively. We therefore repeated our solubilizations of the 263K BHs using the corrected SB recipe, hereafter called SB∗. Indeed, in two independent fractionations (*e.g.* Fig. 1*B*), the marked increases in the detergent concentrations reduced the heterogeneity in our SEC profiles, which now gave more predominant PrP elution in the region of the previously described P1-P2 peaks, *i.e.* peaking slightly after the elution of the 66 kDa BSA marker. However, ultrafiltration of representative SEC fractions again indicated that a 300 kDa high molecular weight cutoff filter reduced seeding activity by > 3000-fold (Table 1).

We then subjected either the SB∗-treated BH or a peak SEC fraction (#50) to SV centrifugation on iodixanol or sucrose gradients (Figure 1*C*, Supplementary Fig. 2). We again saw no evidence of species as small as dimers or tetramers (*i.e.*, ∼55 or 110 kDa, respectively). Instead, we detected much larger PrP aggregates migrating farther than either the 15 to 665 kDa markers or 25 kDa recombinant PrP^C^ 23 to 231 (Fig. 1*C*). We note that prior to SV centrifugation, the SEC fraction was supplemented with iodixanol as described in Experimental procedures to bring its density to that at the top of the continuous gradient, eliminating any abrupt density increase between the sample zone and the top of the linear gradient. This minimized the possibility that PrP particles in the SEC fraction might somehow have reassembled into larger particles due to concentration at a density interface at the bottom of the sample zone. These SV analyses indicated that, even with the updated SB∗ treatment, most of the PrP^Sc^ particles in BHs remained in the form of multimers larger than ∼22-mers.

### Analyses of SB∗-treated mouse 22L prion BH

**Figure 2:**
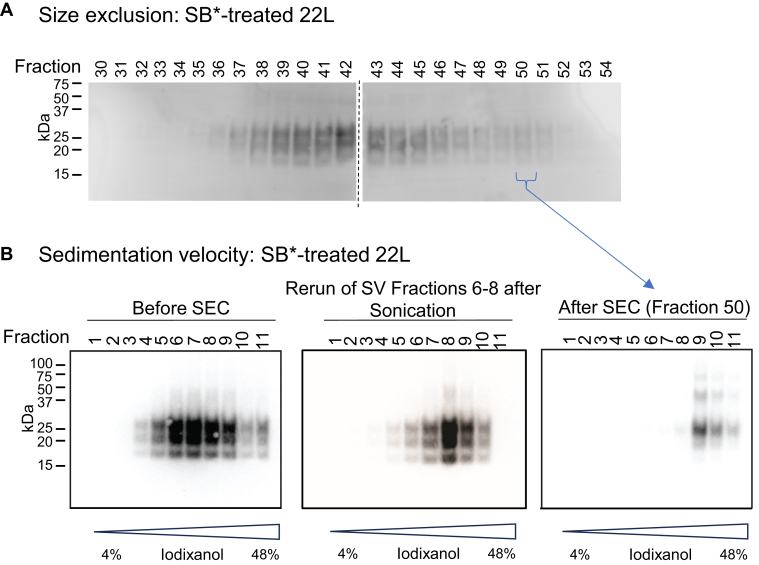
**SEC and SV of SB∗-treated 22LprionBHs**. Brain tissue homogenates from wildtype mice infected with the 22L strain of mouse prions were treated with SB∗ and subjected to SEC (*A*) or SV (*B*) centrifugation on a 4 to 48% iodixanol gradient (*B*, *leftpanel*). SV fractions 6 to 8 were combined, sonicated, and rerun on an SV gradient (*B*, *middlepanel*). SEC fraction 50 from (*A*) was also subjected to SV centrifugation (*B*, *rightpanel*). The *dashed line* in (*A*) indicates the border between individual blots. Similar results were obtained in an independent series of SEC and SV analyses using a second SB∗-treated 22L BH preparation. SEC, size-exclusion chromatography; SV, sedimentation velocity; SB∗, corrected SB; BH, brain homogenate.

Mouse prions have also been reported to disassemble into dimeric-tetrameric oligomers in SB∗ (22). We therefore performed analogous SEC and SV testing of PrP^Sc^ from one of those mouse prion strains, 22L. The PrP^Sc^ in the PK- and SB∗-treated 22L BH eluted in a broad band somewhat earlier than we observed with 263K (Fig. 2*A*) suggesting that the average 22L particle was larger and/or less adherent to the column matrix. Nonetheless, as with 263K, SV analysis of the 22L BH and SEC fraction 50 (which, again, would correspond in elution behavior to the P1/P2 peaks of the earlier study) indicated the predominance of particles that sedimented more rapidly than the 665 kDa protein standard (Fig. 2*B*), with no detectable PrP near the top of the gradients where PrP dimers-tetramers would be expected to remain according to molecular weight markers as shown in Figure 1*C*. Similar results were obtained in an independent series of SEC and SV analyses using a second SB∗-treated 22L BH preparation. We also reran SV fractions 6 to 8 (combined) on SV after resonication but, if anything, the sonication made the PrP^Sc^ particles sediment somewhat faster on average (Fig. 2*B*). Altogether, consistent with the 263K data above, these results provided no evidence of substantial small oligomer formation by 22L PrP^Sc^ in SB∗.

### Particle size approximation using sedimentation analyses without a density gradient

**Figure 3:**
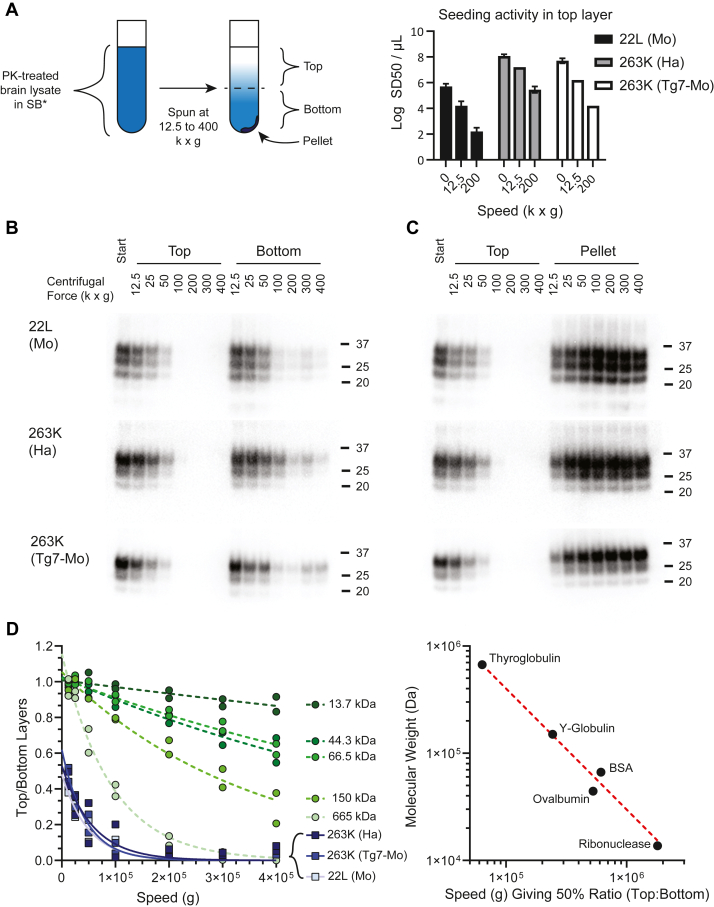
**Sedimentation analysis of SB∗-treated 263K and 22L PrP^Sc^ infected BHs**. *A,* SB∗-treated BHs from 263K infected hamster (Ha) and Tg7 mice expressing hamster PrP (Tg7 Mo), as well as 22L infected wildtype mice (Mo) were analyzed by sedimentation at 12.5, 25, 50, 100, 200, 300, and 400 k x g, after which samples were removed from the *top*, *bottom*, and pellet of each tube (*leftpanel*). The bar plot (*rightpanel*) shows the seeding activity (Log SD50/μl ± SE of undiluted fraction) of each strain in the top layer of the sample after centrifugation at different speeds, which was determined by RT-QuIC. The plotted values represent Log SD50 ± SE estimates generated by classic Spearman-Karber analysis of end point dilution RT-QuIC data from a series of dilutions and multiple replicate wells per sample dilution (8 wells for the unspun samples, 4 wells for the others). Similar results were obtained from an additional set of independent RT-QuIC analyses of the sedimentation fractions of each strain. Immunoblots comparing PrP^Sc^ from the (*B*) *top* and *bottom* as well as the (*C*) *top* and pellet samples from each tube. These blots are representative of duplicate experiments, the results of which are quantitated in (*D*). *D*, quantification of PrP^Sc^ (squares in shades of *purple*) and the protein standards (see Supplementary Fig. 4) thyroglobulin, y-globulin, bovine serum albumin, ovalbumin, and ribonuclease (*circles* in shades of *green*), respectively 665, 150, 66.5, 44.3, and 13.7 kDa. The ratio of protein in the *top* of each sample to that in the *bottom* of each sample (*top/bottom* + pellet) was calculated and is plotted as shown for duplicate spins (*left panel*). *Dashed lines* indicate nonlinear fits to a single-phase decay equation. The speed where 50% of the standard was *left* in the *top* layer, calculated as a single value from the fit to the duplicate datasets, was then plotted as a double log plot (*rightpanel*). RT-QuIC: real-time quaking-induced conversion; SB∗, corrected SB; BH, brain homogenate; PrP^Sc^, scrapie PrP.

To corroborate the above conclusions, and to avoid the possibility that the centrifugation of prion particles through a density gradient might have had a concentrating effect that promoted reaggregation during the SV analyses, we also compared the relative clearance rates of PK- and SB∗-treated prion infected BHs to molecular weight standards in differential centrifugations over a range of speeds (giving average g-forces of 12,500–400,000g) without a density gradient (Fig. 3). After centrifugation of 300 μl of SB∗-treated infected BH, a 150 μl fraction was collected from the top of the gradient with the remaining 150 μl and the pellet collected from the bottom of the gradient (Fig. 3*A* left). Pelleted material, if any, was resuspended in an additional 150 μl of SB∗. RT-QuIC, SDS-PAGE and/or western blot analyses were performed on each fraction. Three different types of prion infected BHs were used that represent models with relatively high (263K in hamsters) and low (263K in Tg7 mice and 22L in RML mice) amyloid plaque accumulations (Supplementary Fig. 3).

The RT-QuIC analyses showed that prion seeding activity in the top zone was depleted by 5-56-fold by the 12.5 k x g spin, and by 500-3000-fold by the 200 k × g spin, depending upon the prion strain/host combination (Fig. 3*A* right). These results, as well as western blot analysis (Fig. 3, *B* and *C*) indicated that the PrP^Sc^ particles in each type of BH sedimented faster than any of the standard proteins, the largest being 665 kDa thyroglobulin (Figure 3*D*, Supplementary Fig. 4). As an index of sedimentation of particles out of the top zone, we calculated the ratio of PK-resistant PrP^Sc^ in the top and bottom zones of the supernatant. With each type of PrP^Sc^, this ratio decreased from a starting value of 1 to ∼0.5 even at the lowest 12.5 k × g spin and continued to drop rapidly with increasing centrifugation speed (Fig. 3*D*). The clearance of PrP^Sc^ from the top zone was accompanied by an increase of PrP^Sc^ in the pellet until, with spins of ≥25 k x g, there was more PrP^Sc^ in the pellet than in the top zone. In contrast, little, if any, of even the largest of the marker proteins (665 kDa) accumulated in a recoverable pellet (Figure 3*D*, Supplementary Fig. 4). This standard, and to a lesser extent, the smaller standards shifted from the top to bottom supernatant zones in a size-dependent manner (Fig. 3*D*), but only at much higher g-forces than was required to do the same with the PrP^Sc^ particles in the brain BHs (Fig. 3, *B* and *D*). For example, at 25 k x g, which had more PrP^Sc^ in the pellet than in the top zone, there was less than a 9% clearance of thyroglobulin (665 kDa) out of the top zone (Figure 3*D*, Supplementary Fig. 4), making it highly unlikely that any much smaller PrP^Sc^ oligomers in the BH were being concentrated in a way that would lead to their reaggregation in the top zone. These results provided further evidence that most of the PK-resistant PrP^Sc^ particles in the SB∗-treated BHs were much larger than 665 kDa. Extrapolating from the linear log-log relationship seen between molecular weight and the g-force required to obtain a top/bottom zone protein ratio of 0.5 using the marker proteins, the majority of PrP^Sc^ particle sizes were estimated to be 2.3 to 3.4 MDa, or the equivalent of 70 to 100 mers, based on their bulk behavior in this analysis (Fig. 3*D*). However, the size heterogeneity of the PrP^Sc^ particles would require that there were also particles that were both larger and smaller than these bulk estimates. Although prion sedimentation might be affected by bound detergent-resistant lipids and the elongated morphologies of PrP^Sc^ fibrils relative to the globular protein standards, these effects are likely to slow sedimentation rates, making these size estimates lower, rather than higher, than the actual values.

### Light scattering and mass photometry analysis

At this point, ultrafiltration and two types of SV analyses each indicated that most of the SB∗-treated particles remained much larger than 300 kDa. This suggested to us that their coelution with much smaller markers (*i.e.* 66 kDa BSA) in SEC was probably due to an artifact such as sticking to the SEC column matrix (see below and (31)). In the previous study (22), the conclusion that the SEC P2 fraction contained PrP dimers was based not only on elution time, but also on static light scattering data indicating the presence of particles of ∼45–55 kDa when the column was loaded with 20 nM purified 263K PrP^Sc^ that had been subjected to SB∗ treatment. However, in attempting to reconcile the results in Figure 1 with previously published work, we considered that at such a low concentration of PrP^Sc^ the light scattering in the fraction might be dominated by detergent micelles of a similar size. This was based on the fact that (1) the SB∗ plus PrP^Sc^ mixture would contain an ∼6 million-fold molar excess of detergent molecules and (2) DDM alone could form ∼8 nm micelles of ∼68 kDa (32). Indeed, even when adjusting the calculations to give a ratio of ∼68 kDa detergent micelles to hypothetical 55 kDa PrP^Sc^ dimers, the particle stoichiometry would be roughly 12,000:1.

**Figure 4:**
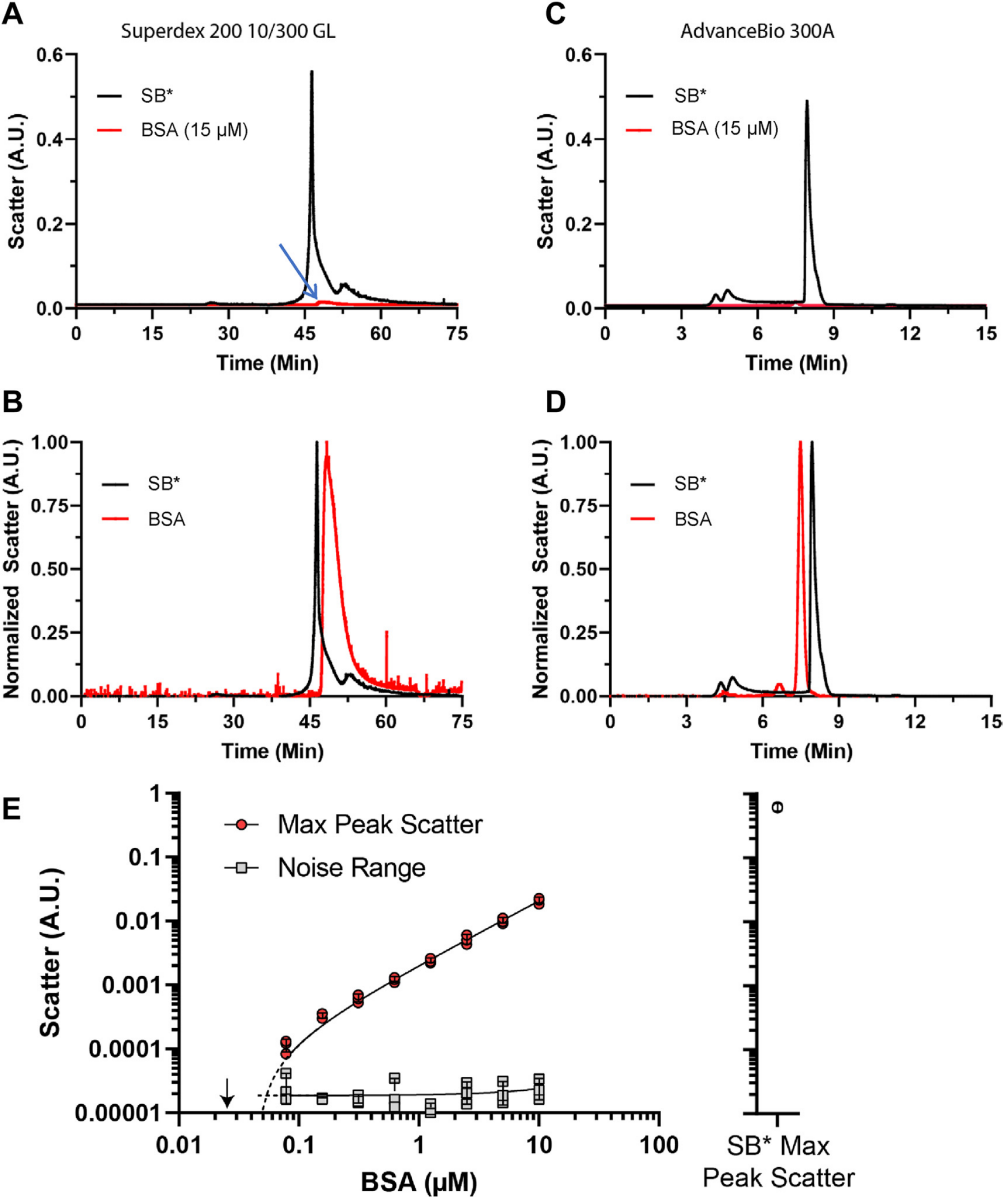
**SEC elution profiles of SB∗ and BSA detected by light scattering using different columns**. *A*, raw traces from Superdex 200 column. *B*, normalized SB∗ and BSA peaks from Superdex column. *C*, raw traces from AdvanceBio 300A column. *D*, normalized SB∗ and BSA peaks from AdvanceBio 300A column. *E*, relative light scattering of BSA SEC peak as a function of BSA concentration relative to the baseline noise level of the detector. For comparison, the scattering maximum of the SB∗ (alone) peak is shown on the same scale on the *right*. Data points are shown from 3 separate SEC runs with each BSA concentration or SB∗ with bars (often no bigger than the data points themselves) indicating the mean ± SD. SEC, size-exclusion chromatography; BSA, bovine serum albumin; SB∗, corrected SB.

We therefore sought to determine detergent micelle size in the DDM + sarkosyl mixture. SB∗, adjusted with water to match its concentration when mixed with BH in the above protocol, was applied to the Superdex SEC column in line with a multiangle light scattering (MALS) detector and eluted as before with a detergent-free mobile phase. A predominant peak eluted shortly after BSA (66 kDa) that had been loaded onto the same column in a separate run (Fig. 4, *A* and *B*). Fractionation on a previously unused AdvanceBio 300A column with similar separation range but a different column matrix composition also showed SB∗ eluting primarily as a peak near where BSA (66 kDa) eluted in an adjacent run (Fig. 4, *C* and *D*), even when diluted as much as ∼5-fold (Supplementary Fig. 5).

To gauge the relative SEC light scattering intensities of SB∗ micelles and BSA, we first loaded BSA onto the column at 15 μM, which is 750-fold more concentrated than the 20 nM PrP^Sc^ loaded on to SEC columns in the previous study (22). Even so, relative to the detergent peak, the BSA peak was barely visible (Fig. 4*A*, blue arrow). Analyses of further dilutions of pure BSA indicated that the minimum concentration giving detectable light scattering above noise was 150 nM or 10 μg/ml (Fig. 4*E*). Thus, light scattering from the ≤ 20 nM of purified P2 fraction of PrP^Sc^ analyzed in the previous study (22) would not be detectable above baseline, at least with our instrument, and in any case would be vastly overwhelmed by scattering from detergent micelles rather than any potential PrP^Sc^ dimers. The presence of micelles in an apparent size range overlapping that of BSA and any putative PrP^Sc^ dimers-tetramers in SB∗ prior to SEC was confirmed by mass photometry, which detected SB∗ micelles from 40 to 250 kDa, peaking at ∼72 kDa (Supplementary Fig. 6). Given that the SEC elution buffer lacked detergent per the previously published protocol, we conclude that the SB∗ detergent micelles eluted from the SEC column at volumes comparable to those of the previously described P1/P2 PrP peaks. Given the vast excess of detergent when mixed with 20 nM PrP^Sc^ as specified (22), the light scattering of the P1/P2 peak of a PrP^Sc^ fractionation (*i.e.*, fractions ∼43–53) should clearly have been dominated by detergent micelles rather than any putative PrP^Sc^ dimers.

### Binding of large PrP^Sc^ particles to the SEC column matrix

**Figure 5:**
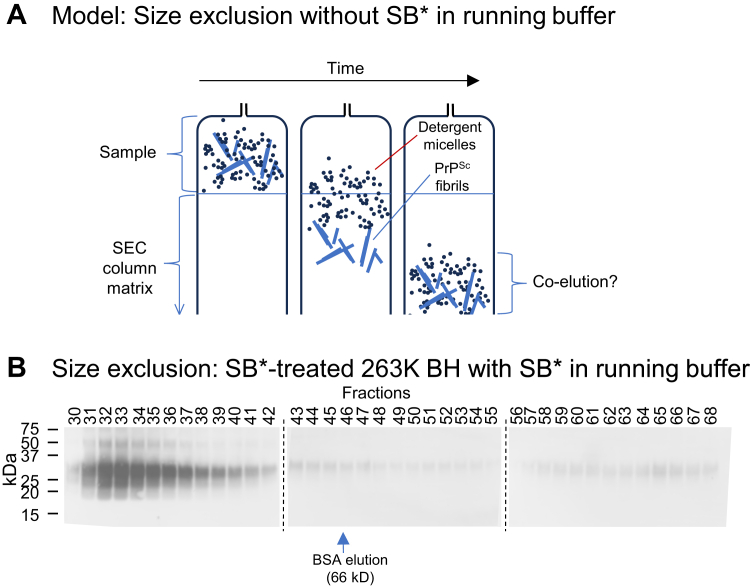
**Inclusion of SB∗ in SEC mobile phase hastens the elution of SB∗-treated 263K PrP^Sc^**. *A*, hypothesis to explain artifactual coelution of larger PrP^Sc^ aggregates with much smaller detergent micelles in the absence of SB∗ in the mobile phase. SB∗-treated, prion-infected BH is loaded onto the SEC column as a mixture of ∼70 kDa detergent micelles and much larger PrP^Sc^ fibrils (*left*). The fibrils begin to move ahead of the micelles because only the latter are able to penetrate the internal pores of the column matrix. However, in the relative absence of detergent the fibrils stick to the column matrix (*middle*) until the micelles catch up with them, resulting ultimately in their coelution in fractions reflecting the size of the micelles rather than the fibrils (*right*). *B*, effect of addition of SB∗ in the SEC mobile phase on the elution of SB∗-treated 263K PrP^Sc^ as detected by western blot. *Arrow*: the fraction at which 66 kDa BSA elutes on this column. Positions of MW markers are shown on the *left*. *Dashed lines* indicate borders between individual blots. A similar elution profile was obtained in an independent replicate SEC separation. SEC, size-exclusion chromatography; BSA, bovine serum albumin; SB∗, corrected SB; BH, brain homogenate; PrP^Sc^, scrapie PrP.

As noted above, we suspected that the main reason for the elution of PrP^Sc^ particles in the apparent dimer-tetramer size range was that they have an affinity for the column matrix. This could delay their elution even if the particles were too large to enter the pores of the SEC matrix. This is a potential artifact of SEC that can make particles appear much smaller than their actual size. In addition, we hypothesized that if > 665 kDa PrP^Sc^ particles are much larger than the ∼40 to 250 kDa detergent micelles in the sample, as indicated by SV centrifugation above (Fig. 1*C*), they would move out ahead of most of the micelles (which are small enough to enter the pores of the matrix) and might stick, at least transiently, to the matrix in the absence of sufficient detergent (Fig. 5*A*). The ensuing wave of detergent micelles might release bound PrP^Sc^ so that the latter arrives in a bolus in the P1/P2 fractions despite being much larger than the monomeric protein standards (*e.g.* 66 kDa BSA) that elute at similar volumes. We reasoned that if SB∗ were included in the SEC mobile phase, then at least some of the PrP^Sc^ in an SB∗-treated BH would elute sooner due to reduced sticking in the continuous presence of detergent micelles. Indeed, this is what we observed. With SB∗ in the SEC mobile phase, the majority of PrP^Sc^ eluted similarly in early fractions ∼31 to 42 in two independent experiments (*e.g.* Fig. 5*B*) *versus* fractions ∼43 to 52 when SB∗ was not in the mobile phase (Fig. 1*B*). Based on the void volume of the column, we estimate that particles that neither adhered to, nor entered, the pores of the column matrix should start eluting near fractions 31 to 32.

**Figure 6:**
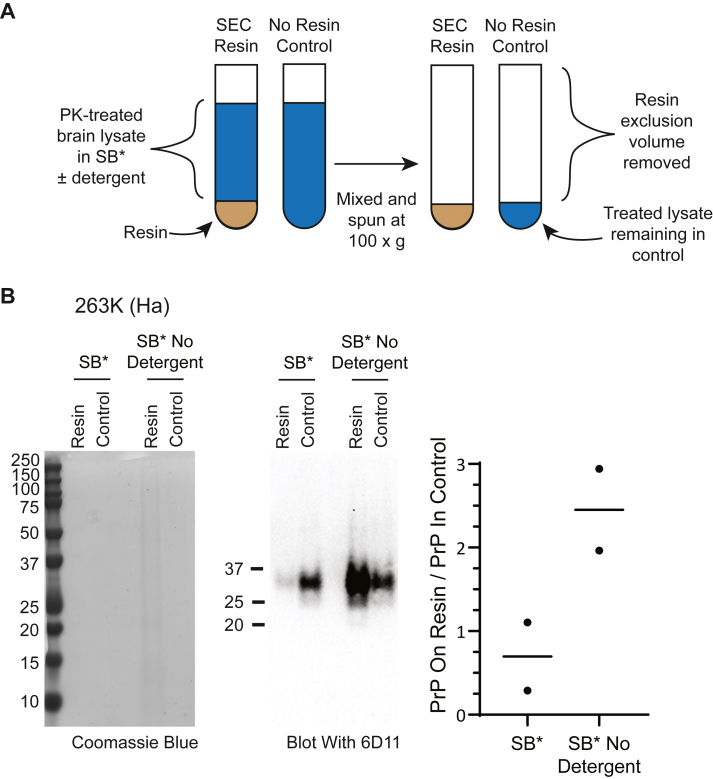
**Increased adherence of 263K PrP^Sc^ to SEC resin in the absence of detergent**. *A*, model demonstrating the experimental setup to determine the amount of PrP^Sc^ that adheres to resin after multiple cycles of dilution, mixing, and centrifugation. Control samples have the same amount of supernatant removed as resin samples allowing the amount of protein that selectively adheres to the resin to be quantified and compared. *B*, representative gels stained for protein with Coomassie blue (*leftpanel*), representative western blots of the same gels developed with the 6D11 antibody (*middlepanel*), and quantitation of the amount of PrP retained by the resin (*rightpanel*) in samples that were, or were not, treated with detergent. Data are given as the ratio of PrP remaining in the resin *versus* the control (PrP on resin/PrP in control). Points shown in the plot are from two separate experiments, and the average of the two samples in each set is indicated by the *black lines*. Molecular weight markers are indicated to the *left* of the gel and blot. SEC, size-exclusion chromatography; PrP^Sc^, scrapie PrP.

Next, we wanted to confirm whether PrP^Sc^ was able to physically adhere to the resin matrix itself by performing a pull-down with the same Superdex 200 resin used in the size exclusion columns (Fig. 6). Preequilibrated resin was mixed with 263K BH treated with SB∗ with and without detergent and compared to a control sample that did not contain resin. All samples were centrifuged and the supernatants removed (Fig. 6*A*). The presence of detergent in SB∗ treated BH prevented adhesion of PrP^Sc^, as well as other proteins, to Superdex 200 resin with ∼2.5X the amount of prion protein associating with resin in the absence of detergent compared to the amount in its presence (Fig. 6*B*). Thus, we conclude overall that most of the PrP^Sc^ aggregates in SB∗ remained much larger than dimers-tetramers. In the absence of SB∗ in the mobile phase, PrP^Sc^ aggregates eluted from this type of SEC column as if they were 55 to 110 kDa dimers-tetramers likely because they adhered to the matrix until the much smaller detergent micelles dislodged them.

### Electron microscopy of SB∗-treated 263K fibrils

**Figure 7:**
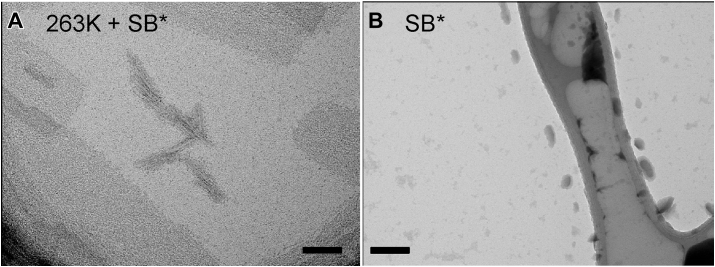
**Negatively stained transmission electron micrograph of PK**-**treatedand SB∗-treated263K PrP^Sc^** (*A*) Purified 263K PrP^Sc^ gently shaken for 4 h at 37 °C in SB∗. (*B*) SB∗ shaken alone. The scale bars represent 200 nm. SB∗, corrected SB; PK, proteinase K; PrP^Sc^, scrapie PrP.

As an orthogonal approach to assessing the effect of PK and SB∗ treatments on 263K prions, we incubated 20 nM purified fibrils in SB∗ as specified previously (22) and then analyzed by negative stain transmission electron microscopy. Despite being scarce (2–3 fibril clusters per EM grid) as expected due to the previously specified very low 20 nM PrP^Sc^ concentration, fibrils that were hundreds of nm long clearly remained after the SB∗ treatment that were not observed in SB∗ alone (Fig. 7).

### Can live cells disassemble 263K prions into small oligomers?

**Figure 8:**
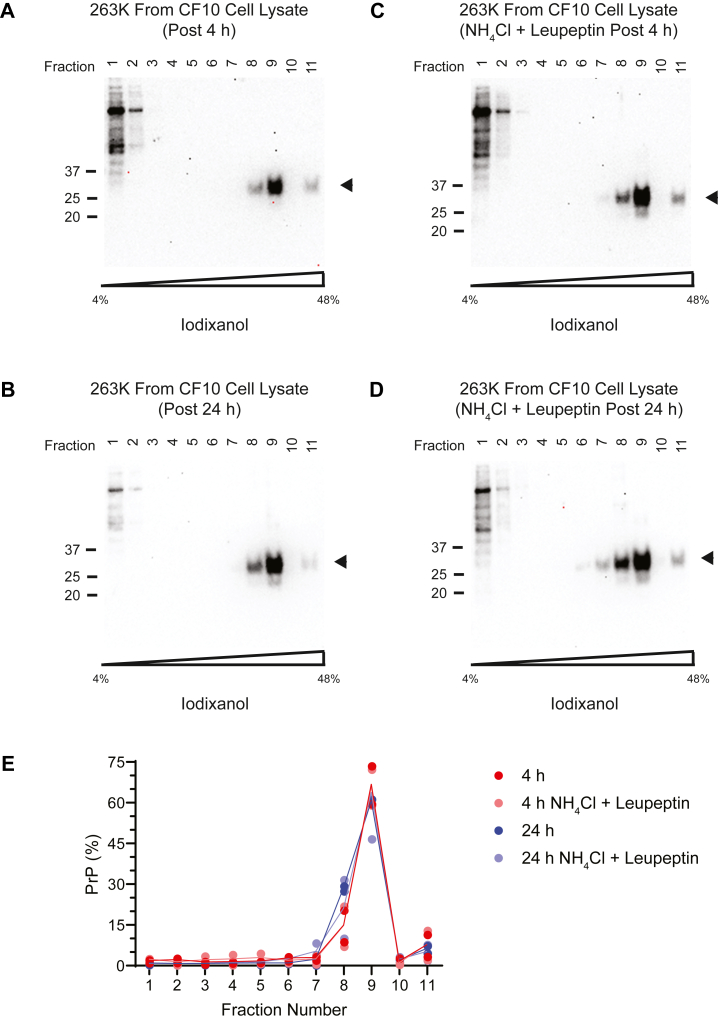
**SV and immunoblotting analysis of CF10 cell homogenates after exposure to 263K-infected BH**. *A*, four hours post exposure. *B*, twenty-four hours post exposure. Four hours (*C*) and 24 h (*D*) post exposure in cells treated with inhibitors of endolysosomal proteases, *i.e.*, ammonium chloride (NH_4_Cl) and leupeptin. The anti-PrP monoclonal antibody 6D11 was used in the immunoblots. *Arrowheads* show the location of PrP^Sc^. *E*, Quantified pixel intensity values are plotted as shown. The identity of the higher MW bands in fractions 1 and 2 in the immunoblots is unclear, but previous work has shown that similar bands in CF10 cells react nonspecifically with the secondary antibody and are not protease resistant (33). SV, sedimentation velocity; BH, brain homogenate; PrP^Sc^, scrapie PrP.

After failing to detect appreciable disassembly of prions into small oligomers in detergents, we revisited the question of whether live cells might be able to disassemble exogenously added prions. In previous work, some of us reported that although murine 22L and 87V PrP^Sc^ can be taken up and fragmented or unbundled into intermediate sized aggregates by CF10 neural cells, most of the PrP^Sc^ remained much larger than dimers and tetramers (33). Here, we performed a similar analysis using 263K PrP^Sc^ because different prion strains have distinct conformations (1, 8, 9, 10, 11, 12) and stabilities (Amyloid Atlas; https://people.mbi.ucla.edu/sawaya/amyloidatlas) and, therefore, may behave differently in the cellular context. BHs (0.25%) from 263K-infected hamsters were applied to CF10 cells, washed, and incubated for 4 or 24 h prior to analyses of cell homogenates by SV on 4 to 48% iodixanol gradients. To probe potential effects of endolysosomal proteases, the cells were incubated with or without ammonium chloride and leupeptin. In both conditions, and at both timepoints, the vast majority of the PrP^Sc^ was detected near the bottom of the gradients in fractions containing particles migrating faster than the 665 kDa protein standard (see Fig. 8). We conclude that CF10 cells were unable to disassemble 263K prions into small, detectable oligomeric units (*e.g.* dimers-tetramers) or, if such oligomers were generated, they were either below the level of detection or rapidly degraded within the cell. This was largely consistent with our previous observations using the murine prion strains 22L and 87V (33), which showed that although those strains were more effectively disassembled into intermediate-sized multimers, there was no evidence of the generation of dimers and tetramers.

## Discussion

### Importance of understanding amyloid fibril fragmentation mechanisms

The fundamental question of whether prions spontaneously disassemble into dimeric-tetrameric units has been debated without resolution for more than 5 decades. The answer affects our consideration of likely mechanisms of prion fragmentation, a key prerequisite for the propagation of prions or any other type of pathological protein aggregate from their origin site to a much wider distribution in the brain. In some cases of acquired prion diseases at least, spreading from peripheral tissues into the brain is required for clinical manifestations of disease. Often this spreading appears to follow routes of neuronal synaptic connectivity (34, 35, 36) in ways that invoke axonal and dendritic transport mechanisms. Indeed, neuritic transport of prions and other PrP aggregates has been well-documented (37, 38, 39). Such intracellular and intercellular spreading mechanisms must be limited profoundly by the size of prion or other amyloid fibrillar aggregates that can be accommodated, necessitating fibril fragmentation or lateral secondary nucleation processes to generate transportable particles as fibrils elongate through templated assembly. However, in the case of PrP prion diseases at least, it seems unlikely that secondary nucleation along the sides of existing fibrils plays a key role in propagation for two reasons. First, the lateral surfaces of most PrP^Sc^ fibril cores are largely inaccessible to macromolecules due to N-linked glycans, glycosylphosphatidylinositol (GPI) anchoring to membranes and/or non-PrP cofactors. Second, the faithful propagation of strain specific PrP^Sc^ conformers implies that it is conformational templating which can only be provided at the ends of prion fibrils, rather than untemplated lateral secondary nucleation, that drives prion growth. Even if only a small proportion of the total PrP^Sc^ needs to be fragmented to allow for intercellular spreading, these arguments emphasize the importance of understanding fibril fragmentation mechanisms not only for understanding prion replication and pathogenesis but also for identifying new therapeutic targets for prion and other proteinopathies.

### A paradoxical history regarding prion disassembly

As mentioned above, the prior literature on the issue of whether prions disassemble spontaneously into small oligomers has long yielded strikingly divergent conclusions. The recent report by Bohl et al., concluded that 4 h SB∗ treatments caused prions to spontaneously disassemble almost entirely into infectious ∼55 kDa dimers and 110 kDa tetramers (22). This study, and the 1982 Prusiner report (21), have provided most of the evidence for efficient autonomous prion disassembly, and, as noted above, were based primarily on SEC and light scattering. Here, we provide the alternative explanation that detection of detergent micelles, and not PrP^Sc^ itself, can account for the apparent presence of dimers and tetramers in PrP^Sc^ separated by SEC and analyzed by light scattering. This explanation reconciles the observed SEC data with the many other types of studies that have indicated that prions remain much larger in nondisinfecting detergents. Thus, we conclude that the vast majority of the available data indicate that PrP^Sc^ prions do not readily and efficiently disassemble into small oligomers under conditions that retain infectivity. This does not mean that small infectious oligomers do not exist, or that they are irrelevant biologically. Of interest in this regard, in the SEC fractions of PrP^Sc^ that were reported previously to be virtually all dimers or tetramers (22), we detected a small proportion (≤∼0.03%) of the seeding activity that passed through 300 kDa ultrafilters and were thus likely to be smaller than the equivalent of a PrP^Sc^ 10-mer. It remains unclear just how small those small seeds get, or whether they existed in the brain, were generated with the SB∗ treatment, or were actually larger particles that were allowed through the ultrafilters due to occasional imperfections. However, our companion manuscript (30) provides molecular dynamics evidence that such prion particles could be stable and retain seeding activity as small as tetramers. From our current biochemical data, we conclude only that once larger assemblies are formed, they do not easily break apart into “elemental bricks” (40) or exist in an equilibrium that markedly favors formation of such dimer-tetrameric units when placed in noninactivating detergent conditions. This conclusion is consistent with the lack of spontaneous fragmentation or disassembly of 2 to 25 mers in the aforementioned molecular dynamics simulations, even at elevated temperatures (30). Nonetheless, even very low proportions of smaller, more mobile prion units might support the dissemination of infection in the host.

## Basis of SEC artifacts with prions

We have provided evidence that PrP^Sc^ tends to bind to the SEC matrix until a bolus of detergent micelles moves through the column dislodging and carrying the much larger PrP^Sc^ particles with them. Thus, our data suggest that it was primarily the size of the micelles, and not PrP^Sc^, that controlled the elution of PrP^Sc^. Importantly, in the analogous Prusiner SEC/gel filtration experiment (21), the PrP^Sc^ was loaded onto the column in 2% sulfobetaine 3-14, but, similarly to the Bohl protocol, the detergent was drastically reduced (by ∼500-fold) in the mobile phase. Therefore, we suspect that, as with our DDM/sarkosyl treatments, the sulfobetaine micelles (which can form ∼30 kDa micelles under at least some conditions) moved as a concentrated wave though the column, dislodging much larger PrP^Sc^ particles as they went.

Additional evidence in earlier work is also consistent with our current interpretation that the detergent micelles determined the SEC elution profile of PrP^Sc^. Notably, one previous study found that if 6 M urea was added to DDM + sarkosyl-treated PrP^Sc^, and then subjected to SEC with the same concentration of urea in the mobile phase, substantial proportions of the PrP^Sc^ particles eluted as if they were much bigger than in dimers or tetramers (40). The authors suggested that 6 M urea had promoted reassembly of PrP^Sc^ fragments. However, given our current results, it seems more plausible that urea reduced PrP^Sc^ binding to the column matrix, allowing it to elute earlier than it would have in the absence of urea.

Why might prions stick to SEC column matrices? Presumably, since the detergent SB∗ components eluted PrP^Sc^ that was bound to the SEC column, hydrophobic interactions play a role. The available high-resolution PrP^Sc^ structures are amyloid fibrils with outwardly facing hydrophobic GPI anchors (when present) that display extended linear arrays of hydrophobic moieties and charges on their flanks (1, 9). Such arrays can mediate multivalent interactions with various types of surfaces which can explain their frequently observed tendencies to bind to each other and to solid surfaces such as SEC column matrices (31).

### How might prions be fragmented, if not spontaneously?

Our work indicates that prion aggregates are not prone to spontaneous fragmentation and likely depend upon cellular process to generate new seeding particles of PrP^Sc^. Previous work has shown that 22L PrP^Sc^ aggregates can be partially fragmented at the cell surface and after endocytosis (33, 41). However, the size of 263K aggregates shifted only marginally after a 24 h exposure to cells (Fig. 8), indicating a strain-dependence to the cell’s ability to disaggregate prion fibrils or clusters thereof. In cellular and tissue environments, PrP^Sc^ fragmentation may be driven by chaperones, proteases, vesicle acidification, membrane dynamics, and interstitial fluid flow. Membrane dynamics may help fragment GPI-anchored PrP^Sc^ (42) on the cell surface (43, 44) and within vesicles (45, 46, 47). The processes of cell division (48), endocytosis (49, 50), and vesiculation (51, 52) can bend and separate membranes, potentially applying stresses to bound PrP^Sc^ fibrils. In addition, the pH shift from 7 to 5 experienced by PrP molecules undergoing endosomal trafficking can destabilize PrP^C^ (53, 54, 55) and PrP^Sc^ (56), which may facilitate membrane mediated fragmentation of the latter. The pH drop may also increase the sensitivity of PrP^Sc^ to activities of chaperones (56) and proteases (45) which can protect cells from nonnative protein aggregates (57). Although cellular PrP^Sc^ loads can be reduced by lysosomal proteases (58), and chaperones like Hsp70 (20, 56, 59) and Hsp110 (60), these same activities can also fragment larger aggregates (20), potentially increasing the concentrations, toxicities, and mobilities of infectious prion particles. Similarly, chaperones have been found to fragment and liberate seeding-competent particles of PrP (20), sup35 (61), α-synuclein (62), and tau (63), indicating that normally protective proteostasis systems may sometimes help to propagate a wide range of disease-associated protein aggregates.

By better understanding how cellular systems facilitate fragmentation of PrP^Sc^ and other pathological aggregates it may be possible to design treatments to reduce their propagation. According to the Amyloid Atlas (https://people.mbi.ucla.edu/sawaya/amyloidatlas), the RML, 22L, and 263K fibril cores have solvation energies (ΔG per residue or per layer) that are on par with, or less stable (less negative), than those of *ex vivo* cross-β fibril cores of α-synuclein (*e.g.* the Lewy fold of Parkinson’s disease and dementia with Lewy bodies) or tau (*e.g.* paired helical or straight filaments of Alzheimer’s disease). On this basis, one might expect that these other types of pathological fibrils might be similarly resistant to spontaneous disassembly into small oligomeric subunits. However, multiple factors besides the core conformation, such as potential influences of poorly resolved peripheral substituents or ligands, may also be impactful.

## Experimental procedures

### Animal care and propagation of prions

The mice and hamsters used in this study were cared for in accordance with the Guide for the Care and Use of Laboratory Animals of the National Institutes of Health. All protocols used for animal care, inoculation, and experiments were reviewed and approved by the Rocky Mountain Laboratories Animal Care and Use Committee (protocol number RML 2016–039). The 22L PrP^Sc^ strain was propagated in RML mice while the 263K PrP^Sc^ strain was propagated in transgenic mice (Tg7) expressing hamster PrP (64) and Syrian golden hamsters. Both mice and hamsters were monitored until they developed clear signs of the onset of prion disease. Animals were then euthanized by isoflurane overdose and brains harvested and stored at −80 °C until use.

### Brain homogenization and protease treatment

Consistent with a previously described protocol (22), brain tissue (∼2 g) from prion-infected rodents (22L in RML mice; 263K in Tg7 mice expressing hamster PrP; or 263K in Syrian golden hamsters) was mixed with 5% glucose and homogenized for 30 s with an Omni TH homogenizer using disposable tips to give a 20% w/v suspension. Proteinase K (PK; Calbiochem, #539480) was added to give a final concentration of 80 μg/ml, followed by incubation at 37 °C for 1 h. The PK was inhibited by adding Pefabloc (Roche, # 11429868001) to 100 μM and placing on ice for 15 min. The PK-treated homogenates were either used immediately or stored at −80 °C prior to further use.

### Treatment with SB∗

As previously described (22) with corrections based on personal communications with the authors, PK-treated BH aliquots (200 μl) were mixed with 225 μl of a solution of 98.2 mM dodecyl maltoside (DDM, Thermo Fisher Scientific #89903); 300 mM NaCl, 10 mM EDTA, 50 mM Hepes, pH 7.4, and vortexed briefly. Then, 57 μl of 20% Sarkosyl was added prior to incubation for 4 h at 37 °C with mild shaking at 300 rpm in a shaking incubator (Eppendorf). After a 5-min spin at 15,000 × g, the largely clarified supernatant was collected and separated from a small pellet.

## SEC of 263K BH treated with PK and SB∗

Consistent with a previously described protocol (22), a 24 ml Superdex 200 10/300 GL gel-filtration column was preconditioned with mobile phase (25 mM Hepes, pH 7.2, 200 mM NaCl, no detergent unless specified) at a flow rate of 0.3 ml/min. Subsequently, 300 μl of cleared SB∗-treated BH was loaded using a sample loop at 0.3 ml/min. Application of the mobile phase was then continued at the same flow rate and 300 μl fractions (1 min/fraction) were collected. The column void volume was 9.8 ml such that particles that were neither included in the pores of the matrix, nor binding to the column, would be eluted near fraction 32. The column was cleaned and decontaminated with 2M NaOH following each run of samples containing PrP^Sc^ and then washed extensively with mobile phase before a new run.

### Ultrafiltration and RT-QuIC analyses of SEC fractions of SB∗-treated 263K BH

Portions of SEC fractions were either left unfiltered or filtered (100 μl) through a 300 kD high molecular weight cutoff spin filter (Pall 300K Nanosep, Cat# OD300C33) with a 4500 × g spin for 5 min. Both the filtrate and unfiltered fractions were analyzed by end point dilution RT-QuIC as described previously (65). Briefly, 2 μl of each sample were seeded into reaction wells neat or after successive 10-fold dilutions into a diluent containing 0.1% SDS in PBS containing 130 mM NaCl with N2 media supplement (Gibco). Analyses of the RT-QuIC data to determine concentrations of seeding units that give 50% positive replicate reactions (SD50) were performed as described (65).

### Superdex 200 SEC resin pull-down of 263K

Superdex 200 resin was initially equilibrated in 1X PBS for 1 h. To two tubes 100 μl of resin (Amersham Biosciences, 17–1043–01) was added before being equilibrated in SB∗ with and without detergent in the separate tubes. Two tubes of controls were setup to contain 100 μl of SB∗ with and without detergent. Next, 800 μl of BH treated with PK and SB∗ that did or did not contain detergent was placed in tubes containing their corresponding detergent with and without resin. Tubes were then gently rocked for 30 min at room temperature before being spun at 100 × g in a tabletop centrifuge for 1 min. From each tube 800 μl was removed and the remaining 100 μl of volume was resuspended in 400 μl of SB∗ that did or did not contain detergent. Tubes were again spun at 100 × g and 400 μl of supernatant was removed. These washes were repeated two more times but with 300 μl additions for a total of around a 1 to 100 dilution of material. The final 100 μl of resin or diluted BH was then mixed with 300 μl of 2x NuPage sample buffer, diluted from a 4x stock (Thermo Fisher Scientific) in 1xPBS. Samples were then vortexed, boiled at 95 °C for 5 min, spun down briefly, and gently centrifuged before being run on a gel.

### Mass photometry

Mass photometry experiments were conducted on a TwoMP instrument (Refeyn) using ultraclean sample carrier slides and sample well cassettes provided by the manufacturer (Refeyn). Prior to the measurement, samples were diluted into 1X PBS (pH 7.4; Corning, REF# 21–040-CV) and 2 μl was applied to the sample well containing 18 μL of PBS. A calibration curve was generated using *B*-amylase, BSA, and thyroglobulin, and data were collected for 1 minute in the medium field of view with the Acquire Software v2024R1.1 (Refeyn). Data were analyzed with Discover MP software v2024R1 (Refeyn).

### Electron microscopy

Purified 263K PrP^Sc^ (3 ml) that was treated at 20 nM with SB∗ as described previously (22) was centrifuged for 45 min at 70 k x g. Supernatant was discarded, and pellet was dissolved in 30 μl SB∗. Briefly, samples were vortexed and adhered to glow-discharged ultrathin carbon on lacey carbon 400 mesh copper grids (Electron Microscopy Sciences) for 1 h. Samples were lightly blotted, followed by a ddH_2_O rinse, lightly blotted, and finally stained with Nano-W. Grids were imaged in a HT7800 (Hitachi) transmission electron microscope operating at 80 kV. Micrographs were acquired on an XR-81 camera (Advanced Microscopy Techniques).

### Iodixanol gradient separation of prion and standard proteins

Linear iodixanol gradients ranging from 0 to 10, 4 to 48, and 10 to 25% with or without SB∗ were created from dilutions of a 60% iodixanol solution in OptiPrep density gradient medium (Sigma-Aldrich), that were stacked within and then linearized by a Gradient Master 108 (BioComp). Protein standard mixtures (Sigma-Aldrich), BSA, recombinant PrP, BHs, and cell samples were loaded onto gradients in iodixanol solutions matching the lowest iodixanol percentage for the gradient onto which it would be loaded (0, 4, and 10% iodixanol in 1X PBS). All dilutions were carried out in 1x PBS and gradients had a total volume of 4.5 ml while samples had a volume of 0.5 ml. Gradients were spun at 200 k x g in an SW55 TI rotor (Beckman Coulter) for 1 h at 4 °C. After centrifugation, 11 x 450 μl fractions were from the top of each gradient. Each fraction was later prepared and analyzed by SDS-PAGE and western blot.

### Sedimentation analysis of protein standards and prion protein

BHs and protein standards that were respectively treated with or diluted in SB∗ were centrifuged at 12.5, 25, 50, 100, 200, 300, and 400 k x g in TLA 100.1 rotor (Beckman Coulter) for 30 min. The total size of each sample was 300 μl. After centrifugation, top and bottom halves (150 μl each) of the supernatant from each sample were removed and placed in separate tubes. The pellet was resuspended in 150 μl of 1x PBS before being placed in a separate tube. Samples were then prepared and analyzed *via* western blot and SDS-PAGE.

### SEC-MALS analysis of SB∗

The size of protein standards and micelles in the SB∗ detergent solution, as well as in solutions containing only one of the detergents, was evaluated using a Wyatt Dawn 8 SEC-MALS equipped with either an AdvanceBio SEC 300 Å 2.7 μm by 7.8 x 300 mm column or a Superdex 200 Increase 10/300 GL column. Light scattering from SEC-MALS was evaluated with ASTRA 7.3.2.21 software calibrated with 1 mg/ml BSA. The light scattering profiles were directly compared in some plots to demonstrate the vast difference in intensities between the light scattering of SB∗ and 15 μM BSA, a standard eluting near SB∗ micelles. Also, the elution profiles were normalized by their maximal intensities to better compare elution times. Data were imported into Prism GraphPad Software (version 8.0.2) for analysis and figure generation.

### Exposure of CF10 cells to 263K PrP^Sc^

BHs of 263K were diluted to 0.25% BH in Opti-MEM supplemented with 10% fetal bovine serum and 0.1 mg of both penicillin and streptomycin. The BH laden medium was then split and half of the medium was mixed with 10 mM ammonium chloride and 0.2 mg/ml leupeptin, while the other medium was given equivalent volumes of 1x PBS. The different media were gently placed over CF10 cells that do not express PrP^C^ (66). CF10 cells were grown to ∼95% confluency in 6-well plates and each well received 1.5 ml of media. CF10 cells were then incubated with each medium for 4 h and 24 h at 37 °C and 5% CO2 before the medium was removed, cells washed in Opti-MEM, and then lysed on the plate with 1 ml of lysis buffer (0.5% Triton X-100, 0.5% sodium deoxycholate, 5 mM Tris–HCl pH 7.4, 150 mM NaCl, and 5 mM EDTA). Cell homogenates were gently mixed and then spun at 5 k x g in a tabletop centrifuge for 2 min. Supernatants were then removed and analyzed by iodixanol gradient.

### SDS-PAGE and western blots

SV or SEC samples were run slightly differently on SDS-PAGE and western blots. SV samples were mixed with sample buffer to a final concentration of 1.5x sample buffer before being denatured by incubation at 95°C for 5 min and then run over a 10% Bis-Tris (NuPAGE) gel. Gels containing non-PrP^Sc^ standards were then stained with 1x Imperial Stain (Thermo Fisher Scientific) for ∼1 h and destained before being scanned on an Epson flatbed scanner. Gels containing PrP^Sc^ samples were blotted onto polyvinylidene difluoride (PVDF) Immobilon-P membranes (EMD Millipore) using a Mini Blot Transfer Cell system (Bio-Rad). Membranes were then blocked by incubation for 1 h in 1xTBST (10 mM Tris–HCl (pH 8.0), 150 mM NaCl, and 0.05% Tween 20) containing 5% powdered milk. Membranes were rinsed twice in 1xTBST before being probed for 1.5 h with the 6D11 primary antibody directed at PrP residues 93 to 109 (BioLegends) diluted 1:7000 in 1xTBST, washed in 1xTBST, and then reprobed for 1 h with a horseradish peroxidase conjugated sheep anti-mouse secondary antibody (GE Amersham) diluted to 1:25,000 in 1xTBST. After a quick rinse with deionized water, membranes were mixed with ECL Plus reagent (GE Healthcare Life Sciences). Membranes were developed for 1 min before being imaged on an iBright gel imager (Thermo Fisher Scientific).

For western blots of SEC fractions, fractions were mixed with an equal volume of 2X SDS-PAGE buffer to give final concentrations of 62.5 mM Tris pH 6.8, 5% SDS, 2.5% glycerol, 4% β -mercaptoethanol, 0.02% bromophenol blue, 3 mM EDTA, and 4 M urea, boiled for 5 min, resolved on 10% Bis-Tris, NuPAGE gels (Invitrogen), and transferred to Immobilon-P PVDF membranes (EMD Millipore) using an iBlot system (Invitrogen). PrP bands were then developed on the membranes using either monoclonal PrP antibody 3F4 at 1:10,000 and goat anti-mouse IgG alkaline phosphatase conjugate at 1:10,000 (Calbiochem, Sigma-Aldrich #DC05L) for the 263K blots or the polyclonal rabbit antiserum R30 raised against a synthetic peptide homologous to mouse PrP residues 89 to 103 (67) at 1:5000 with Goat anti Rabbit-AP secondary at 1:5000 for 22L blots. The blots were developed with AttoPhos substrate (Promega). Digital images of the developed PVDF membranes were then captured on the Typhoon FLA 9500 scanning system (GE Healthcare).

### Quantification and analysis of blots

Bands on gels and western blots were quantified using UN-SCAN-IT software (version 7.1; Silk Scientific Corporation). The “Segment Analysis” tool was used to sum all pixels in analysis regions where bands were found or regions near bands that were used for background correction. Background corrections were performed by subtracting the pixel sum of band containing regions from the pixel sum of regions that did not contain bands. For analysis of iodixanol gradients, background corrected pixel sum values were converted to a percentage of total prion protein by dividing the pixel sum in each fraction by the total sum of all background corrected pixel sums in all fractions and multiplying by 100. For analysis of sedimentation assays, the background-corrected pixel sum of protein in the “top” layer was divided by the pixel sum of protein bands in the “bottom” layer and “pellet” samples (top/(bottom + pellet)). Respective percentages of total PrP and ratios of protein across centrifuge tubes were then plotted in Prism GraphPad Software (version 8.0.2), which was used for both data management, figure creation, and further analysis. Ratio data from sedimentation assays were fitted with a least square curve in Prism GraphPad, which also calculated the EC50 values of each fit. The EC50 values of each standard were plotted on a double log plot, which was fit to a double log line in prism. The equation from this standard line was used to approximate the size of PrP^Sc^ in sedimentation assays.

### Immunohistochemistry

Brain collection, fixation, embedding, and immunohistochemistry staining for prion protein using anti-prion antibody EP1802Y (GeneTex GTY61655) has been described previously (68). Brains were removed from uninfected and 263K prion infected Syrian hamsters or tg7 mice at end-stage disease.

## Data availability

All original data associated with this paper, including original images of gels, blots, electron micrographs, *etc.*, whether show explicitly or not in the figures or supporting information, are available from Byron Caughey (bcaughey@nih.gov) upon request.

## Supporting information

This article contains supporting information.

## Conflict of interest

The authors declare that they have no conflicts of interest with the contents of this article.
