# Supplementary material for: Evidence against efficient spontaneous disassembly of prions into small oligomers
Source: J Biol Chem. 2025 Jun 21;301(8):110411. doi: 10.1016/j.jbc.2025.110411 (PMC12301739; doi:10.1016/j.jbc.2025.110411)
Supplement: Supporting Information [file mmc1.pdf]

Evidence against efficient spontaneous disassembly of prions into small oligomers

Daniel Shoup‡[1], Andrew G. Hughson‡[1], Brent Race[1], Parvez Alam[1], Daniel Dulebohn[2], Suzette A. Priola[1]*, Byron Caughey[1]*

SUPPORTING INFORMATION

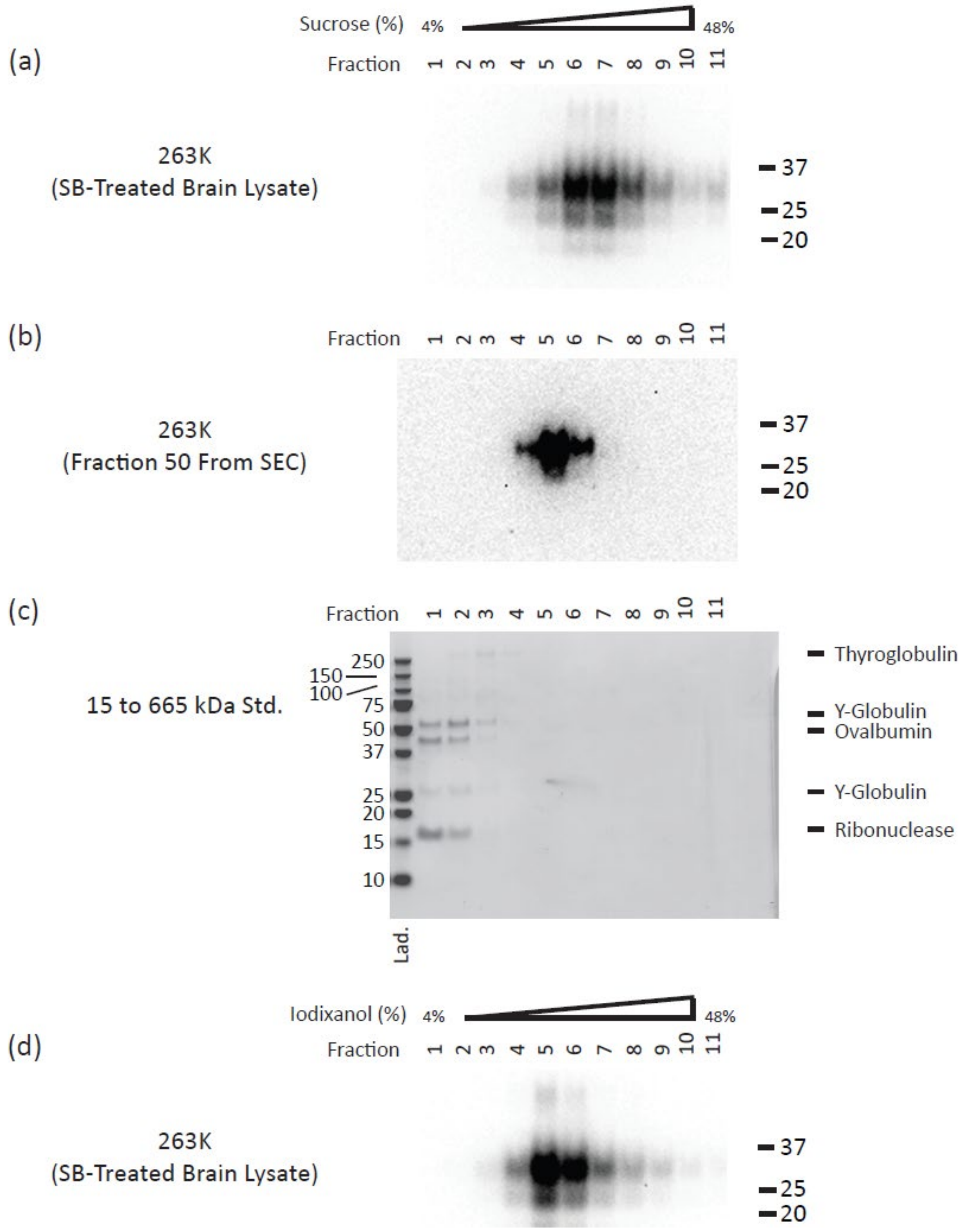

Supplementary Figure 1. **Comparison of SB-treated 263K BH sedimented over sucrose and iodixanol.** Hamster brains infected with 263K prions were treated with SB and analyzed by (a) sucrose gradient before and (b) after SEC separation. (c) Standards ranging from 15 to 600 kDa were also run over sucrose gradient for size comparison. (d) Samples of 263K BH were centrifuged over iodixanol gradients. Each gradient was broken into 11 fractions that were analyzed on SDS PAGE gels alongside molecular weight standards as shown.

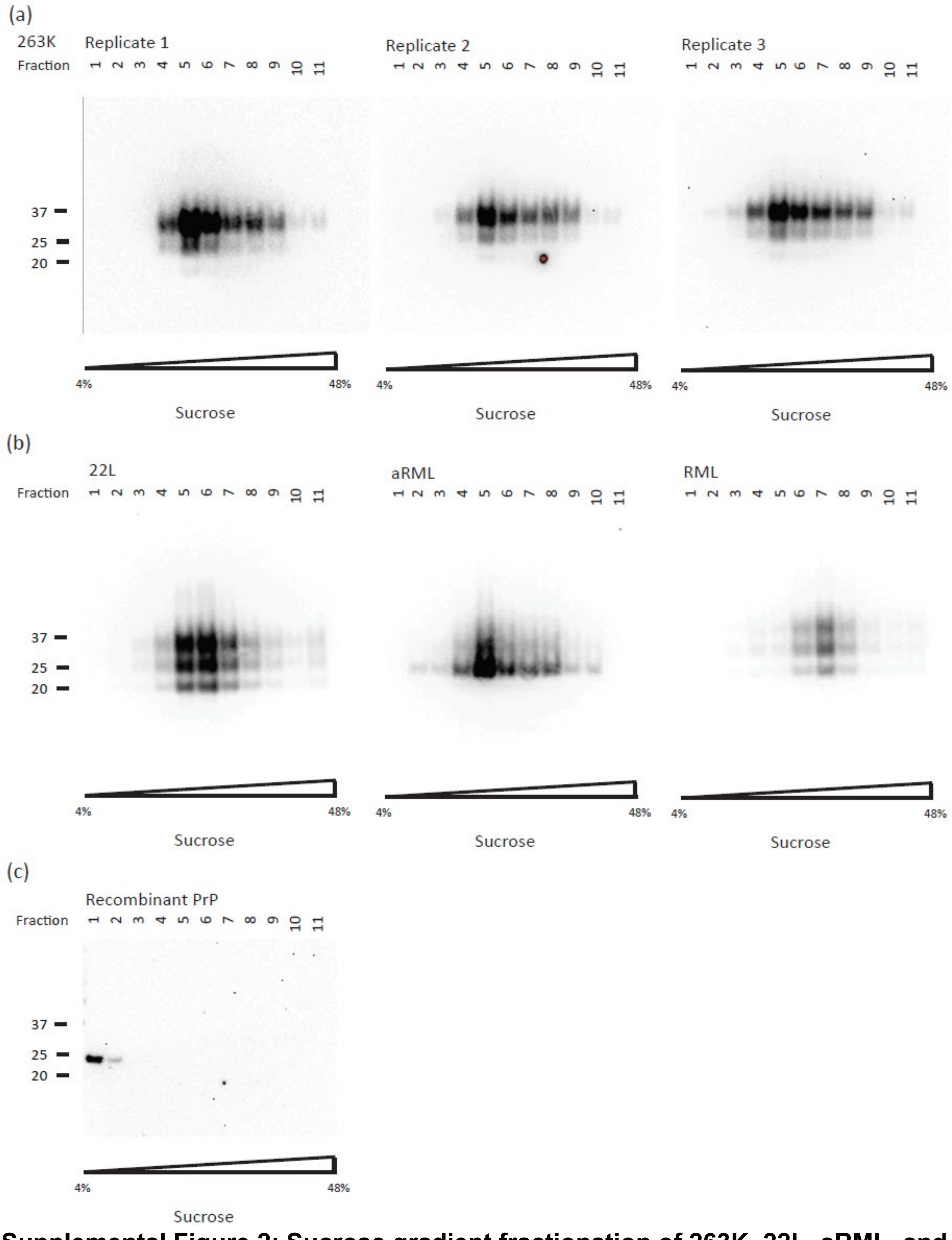

Supplemental Figure 2: Sucrose gradient fractionation of 263K, 22L, aRML, and RML reveals that SB* treatment of PrP^{Sc} does not produce small oligomers. Anti-PrP Western blots of sucrose gradient fractionated SB*-treated (a) hamster 263K BH, in triplicate, as well as individual runs of (b). 22L, aRML, RML, and (c). recombinant PrP are shown. Sucrose gradients were set up as continuous 4 to 48% gradients. Positions of SDS-PAGE MW markers are shown on the right.

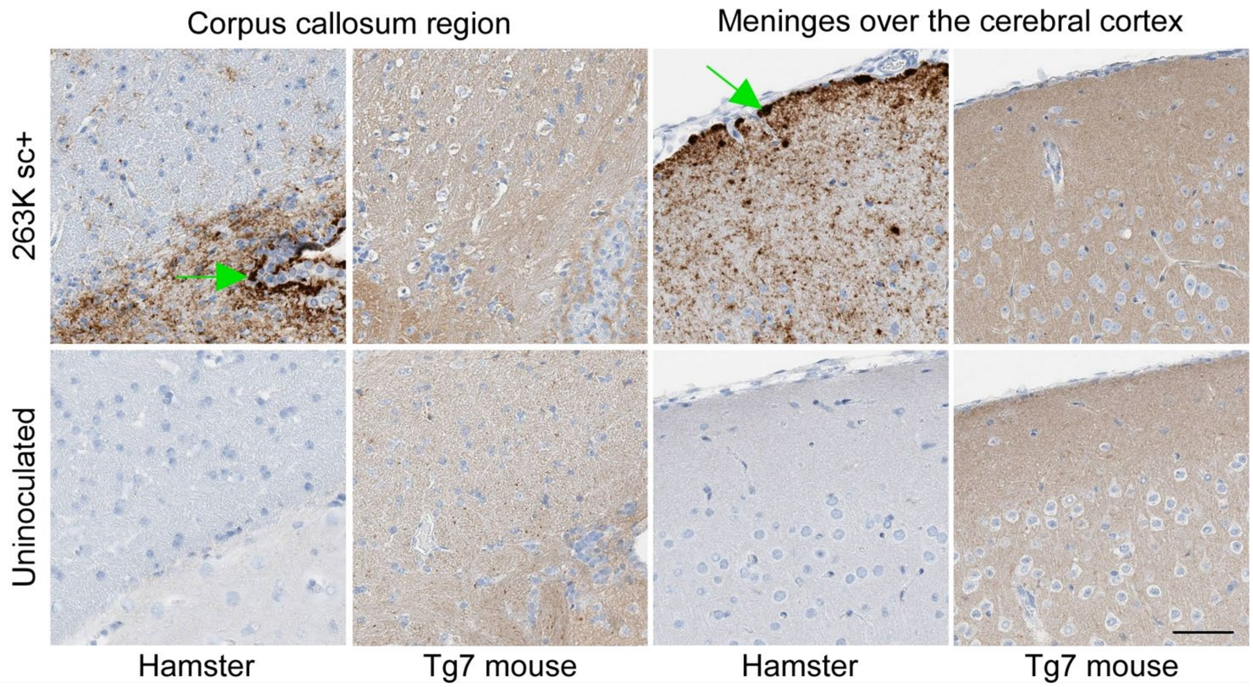

Supplementary Figure 3. **Relative plaque loads in 263K-infected hamsters versus Tg7 mice.** Immunohistochemistry (IHC) for prion protein using anti-prion antibody EP1802Y. The brain regions shown are described at the top of the figure, the infection status is shown on the left, and the species (Syrian golden hamster or tg7 mice) is shown below each column. The green arrows indicate small PrP^{Sc} plaques present in 263K infected hamsters, particularly near the meninges, corpus callosum, and ependymal cells of the ventricles. PrP^{Sc} deposition in other locations of the hamster brain is primarily as diffuse (synaptic) or as smaller puncta. Tg7 mice have high expression levels of PrP^{C} (Uninoculated, light brown hue) but minimal PrP^{Sc} deposition detectable by IHC, even at end stage disease. The scale bar in the lower right is 50 µm and applies to all panels.

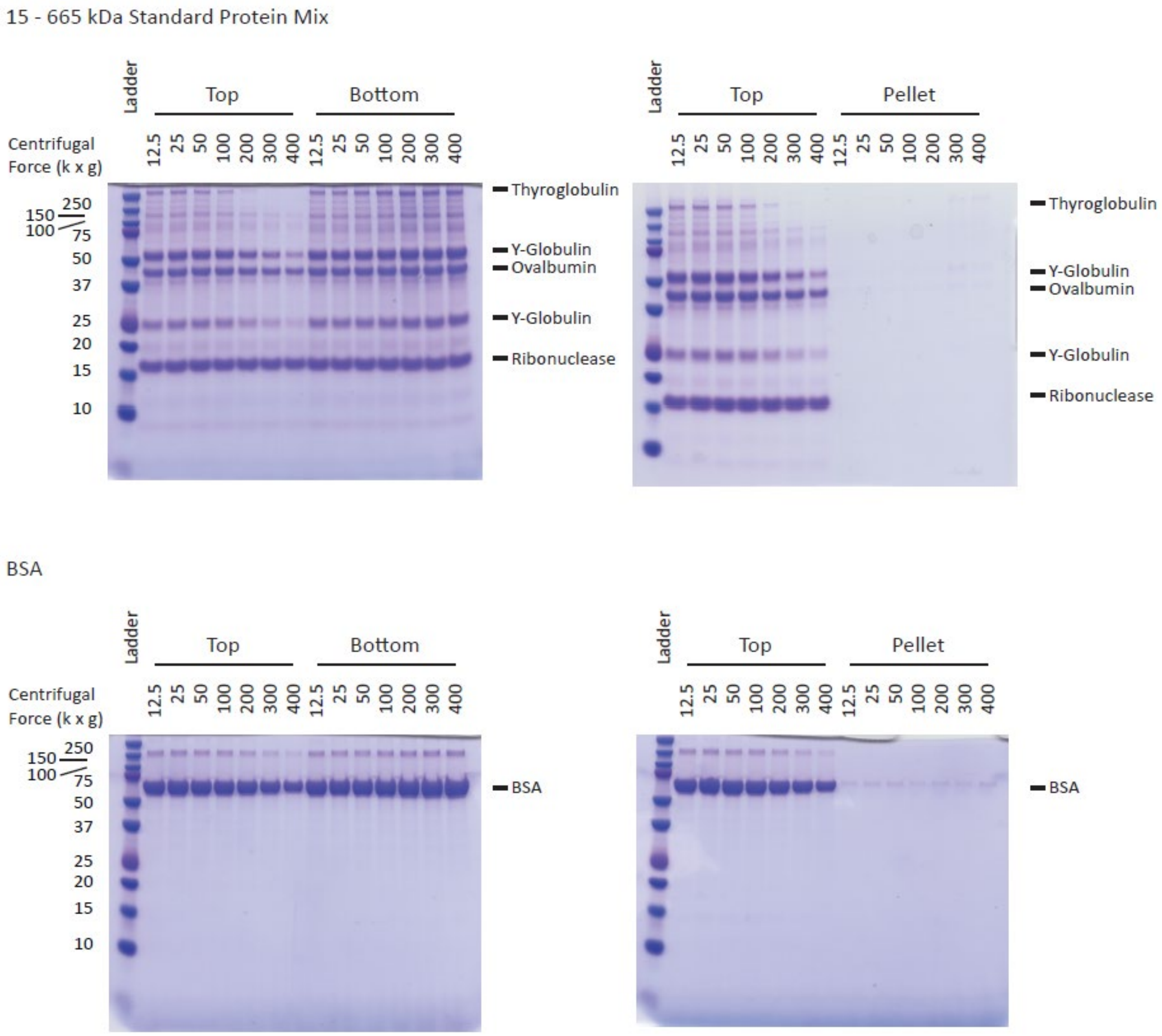

Supplementary Figure 4. **Sedimentation analysis of protein standards**. A mixture of protein standards (upper panels) thyroglobulin (665 kDa), y-globulin (150 kDa), ovalbumin (44.3 kDa), and ribonuclease (13.7 kDa) as well as bovine serum albumin (BSA, 66.5 kDa, lower panels) were analyzed by sedimentation at 12.5, 25, 50, 100, 200, 300, and 400 k x g. Samples were then removed from the top, bottom and pellet of each tube (see graphic at the top of Figure 6) and analyzed by SDS-PAGE. Shown are representative gels of each standard, indicated to the right of each gel. Samples from the top were compared to samples from the bottom (left panels) and pellet (right panels).

SB* dilution by SEC MALS

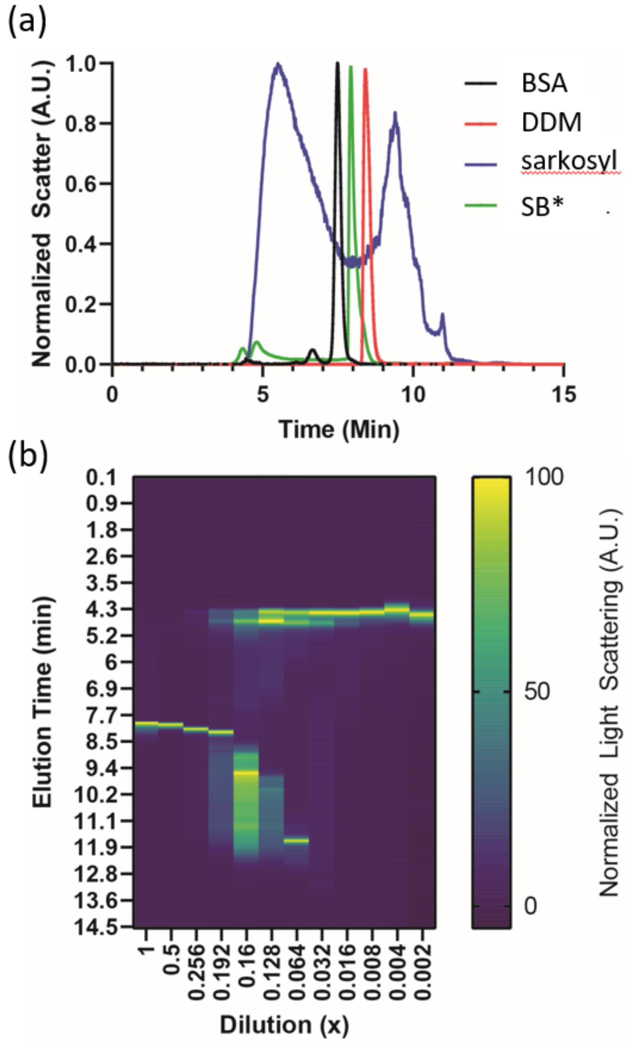

Supplementary Figure 5. SEC-MALS analysis of SB* detergent micelles. (a). SB* (DDM + sarkosyl), DDM, sarkosyl, and BSA were fractionated on a AdvanceBio 300A column in line with a MALS detector without detergent in the mobile phase. The peak light scattering signals were normalized between the different samples. (b). Comparison of the elution profiles of the designated dilutions of SB*. The micelle elution patterns were similar across 1x-0.26x SB* concentrations (with 1x being the concentration in SB*-treated BHs), i.e., eluting slightly after the 66-kDa BSA marker as noted above. With 0.192x-0.064x SB* concentrations, micelle subpopulations emerged that eluted faster and slower than BSA. With further dilutions, the more rapidly eluting population became predominant. Thus, the predominant micelle size remained largely consistent through several-fold dilutions of SB* although more extreme dilutions lead to changes in elution behavior and therefore, presumably, micelle structure. Interestingly, the elution profile of DDM alone was slightly slower than that of the DDM + sarkosyl mixture in SB*, but sarkosyl alone gave a much broader elution profile (a).

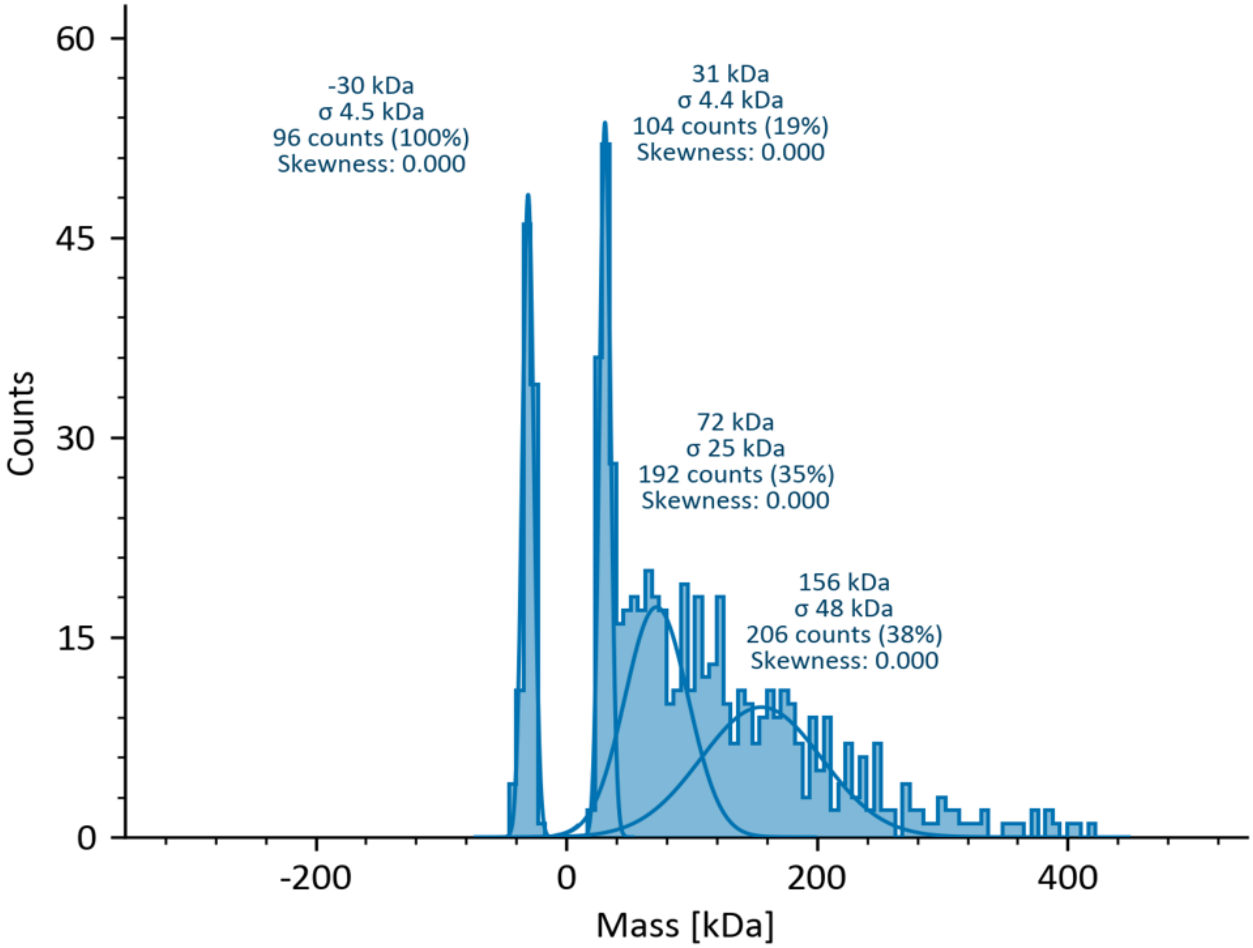

Supplementary Figure 6. **Mass photometry measurement of SB* detergent micelle size.** SB* was assayed at 1:1000 dilution due to artifactual interferences with this analysis when undiluted. The 30-31 kDa peaks that are mirrored on both the + and – sides of the mass axis represent commonly observed artifacts of mass photometry at the lower limit of the detectable range of particle sizes (i.e., ~30 kDa). The remaining counts at larger masses correspond to SB* components which were dominated by sarkosyl and DDM. Two repeat analyses yielded similar plots.
